# Role of plant relatedness in plant–soil feedback dynamics of sympatric *Asclepias* species

**DOI:** 10.1002/ece3.9763

**Published:** 2023-01-24

**Authors:** Eric B. Duell, James D. Bever, Gail W. T. Wilson

**Affiliations:** ^1^ Kansas Biological Survey & Center for Ecological Research Lawrence Kansas USA; ^2^ Department of Ecology & Evolutionary Biology University of Kansas Lawrence Kansas USA; ^3^ Department of Natural Resource Ecology & Management Oklahoma State University Stillwater Oklahoma USA

**Keywords:** arbuscular mycorrhizal (AM) fungi, *Asclepias*, congener, mutualists, phylogenetic relatedness, plant–soil feedback (PSF)

## Abstract

Plants affect associated biotic and abiotic edaphic factors, with reciprocal feedbacks from soil characteristics affecting plants. These two‐way interactions between plants and soils are collectively known as plant–soil feedbacks (PSFs). The role of phylogenetic relatedness and evolutionary histories have recently emerged as a potential driver of PSFs, although the strength and direction of feedbacks among sympatric congeners are not well‐understood. We examined plant–soil feedback responses of *Asclepias syriaca*, a common clonal milkweed species, with several sympatric congeners across a gradient of increasing phylogenetic distances (*A. tuberosa*, *A. viridis*, *A. sullivantii*, and *A. verticillata*, respectively). Plant–soil feedbacks were measured through productivity and colonization by arbuscular mycorrhizal (AM) fungi. *Asclepias syriaca* produced less biomass in soils conditioned by the most phylogenetically distant species (*A. verticillata*), relative to conspecific‐conditioned soils. Similarly, arbuscular mycorrhizal (AM) fungal colonization of *A. syriaca* roots was reduced when grown in soils conditioned by *A. verticillata*, compared with colonization in plants grown in soil conditioned by any of the other three *Asclepias* species, indicating mycorrhizal associations are a potential mechanism of observed positive PSFs. This display of differences between the most phylogenetically distant, but not close or intermediate, paring(s) suggests a potential phylogenetic threshold, although other exogenous factors cannot be ruled out. Overall, these results highlight the potential role of phylogenetic distance in influencing positive PSFs through mutualists. The role of phylogenetic relatedness and evolutionary histories have recently emerged as a potential driver of plant–soil feedbacks (PSFs), although the strength and direction of feedbacks among sympatric congeners are not well‐understood. Congeneric, sympatric milkweeds typically generated positive PSFs in terms of productivity and AM fungal colonization, suggesting the low likelihood of coexistence among tested pairs, with a strength of feedback increasing as the phylogenetic distance increases.

## Graphical abstract

The role of phylogenetic relatedness and evolutionary histories have recently emerged as a potential driver of plant‐soil feedbacks (PSFs), although the strength and direction of feedbacks among sympatric congeners is not well understood. Congeneric, sympatric milkweeds typically generated positive PSFs in terms of productivity and AM fungal colonization, suggesting low likelihood of coexistence among tested pairs, with strength of feedback increasing as phylogenetic distance increases.

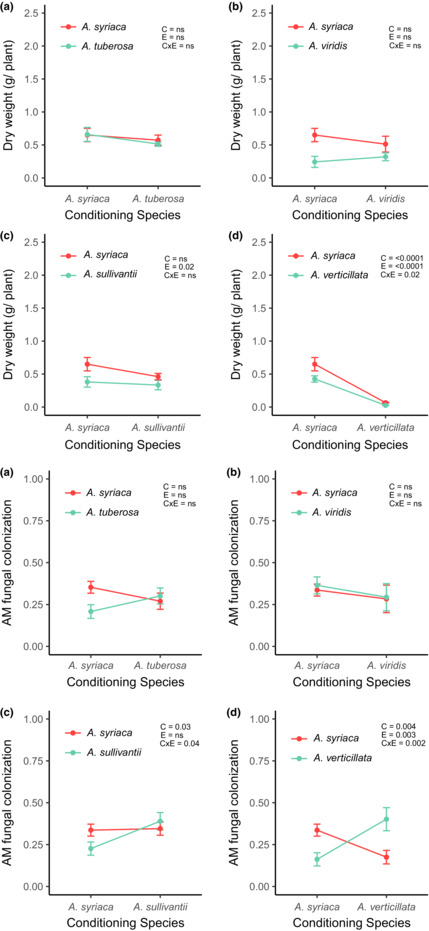

## 1 INTRODUCTION

Interactions between plants and associated soils play an important role in the shaping and maintenance of plant communities (Bever et al., 2015; Mangan, Schnitzer, et al., 2010). Plants actively exude phytohormones, sugars, and other compounds, which directly and indirectly influence the biotic and abiotic properties of rhizosphere soil (Bever, 1994; De Long et al., 2018; Meiners et al., 2017). The effects of plants on local soil properties, along with subsequent reciprocal interactions, are collectively known as plant–soil feedbacks (PSFs) (Bever et al., 2010). Plant–soil feedbacks span a continuum from negative to positive, and the direction and magnitude of the feedback are often determined by the presence or absence, as well as abundance, of certain soil biota (Crawford et al., 2019). Negative PSFs occur when plant growth is reduced in soil previously occupied by plants of the same species (conspecific‐conditioned), compared to growth in soil previously occupied by a different species (heterospecific‐conditioned). Alternatively, positive PSFs occur when plants produce greater biomass in soil conditioned by conspecifics, relative to heterospecific‐conditioned soil. In plant communities, negative PSFs encourage heterogeneity and coexistence between heterospecifics; thereby promoting greater plant diversity, whereas positive PSFs promote monotypic stands, with resultant reductions in plant diversity (Bever et al., 1997; Mack et al., 2019). Plant species within native, co‐evolved communities typically exert negative PSF, where the species generally performs better in soils conditioned by heterospecifics, which facilitates greater overall plant diversity (Crawford et al., 2019; Meiners et al., 2017; Stein & Mangan, 2020). However, a number of factors influence the magnitude and direction of PSF dynamics between species within a localized plant community, with tremendous implications for community‐level interactions contributing to heterogeneity. Elucidation of mechanisms driving these interactions is critical for further understanding of competitive dynamics that contribute to observed community patterns.

One such mechanism may be plant relatedness. Closely related species are more likely to share common traits or characteristics due to a longer shared evolutionary history, relative to species that diverged earlier in history (Pagel, 1999). Over the last decade, a growing body of literature suggests that phylogenetic relationships influence plant–soil interactions (Anacker et al., 2014; Crawford et al., 2019; Hoeksema et al., 2018; Kempel et al., 2018; Segnitz et al., 2020; Wandrag et al., 2020). Although diverse, native plant communities typically display high levels of phylogenetic diversity with representatives from multiple genera, families, and orders, many ecosystems support suites of sympatric congeners, and the role of PSFs among co‐occurring congeners is poorly understood (Wilschut et al., 2019). While some studies have found no relationship between phylogenetic distance and PSF (Mehrabi & Tuck, 2014; Wilschut et al., 2019), many others have found considerable support for phylogenetic relatedness influencing PSF dynamics (Anacker et al., 2014; Crawford et al., 2019; Hoeksema et al., 2018; Kempel et al., 2018; Wandrag et al., 2020). However, PSF can be driven by several mechanisms, some of which may be differentially influenced by phylogenetic relatedness of host plants. For instance, the degree of host specificity of soil pathogens and mutualists may depend upon phylogenetic scale, with pathogens being more specialized in lower‐order taxa [e.g. within genera (Wang, Hoffland, et al., 2019; Wang, Jiang, et al., 2019)]. Strong phylogenetic conservatism of host‐specific pathogens suggests that many plant species should exhibit strong negative PSF when soil is conditioned by phylogenetically distant plant species, due to the lack of shared antagonists (Collins et al., 2020; Gilbert & Webb, 2007; Parker et al., 2015). Alternatively, PSFs may be driven by the presence or absence of mutualists, which typically show greater specificity at higher‐order taxonomic levels [e.g. family and order (Wang, Hoffland, et al., 2019; Wang, Jiang, et al., 2019)]. For example, plants that do not share mutualistic cohorts will likely exhibit positive PSFs if each plant species benefits equally from the host‐specific mutualists (Hoeksema et al., 2018; Reinhart et al., 2012). In this scenario, if phylogenetically distant species are less likely to share mutualists due to earlier divergences, relative to more closely related species, then PSFs will likely become more positive as the phylogenetic distance increases (Crawford et al., 2019). The vast majority of studies examining the role of evolutionary history in plant–microbial interactions do so at very coarse phylogenetic scales, incorporating relatively large numbers of species from multiple genera or families (Crawford et al., 2019; Hoeksema et al., 2018). Negative feedbacks have been shown to predominate at such coarse scales (Crawford et al., 2019), consistent with deeply conserved plant traits related to pathogen defense. Whether variation in mutualists becomes more important at finer phylogenetic scales remains unknown.

Milkweeds (*Asclepias*) are a diverse genus of plants in the milkweed and dogbane family (Apocynaceae), with 130 of the estimated 400 species found in North America. Milkweed research has increased dramatically over the past two decades, due in part to its importance as the primary food source of monarch (*Danaus plexippus* L.) larvae. Only recently have researchers started to explore the complex relationships between milkweeds and soil symbionts, particularly arbuscular mycorrhizal (AM) fungi. Even less research has been conducted assessing PSF that may be occurring between different milkweed species, with only one study being published to date (Snyder & Harmon‐Threatt, 2019). In the grasslands of central North America, several *Asclepias* species can be observed growing within relatively close proximity to one another. Common sympatric species in North American prairies include *Asclepias syriaca* L. (common milkweed), *A. tuberosa* L. (butterfly milkweed), *A. viridis* Walter (green antelopehorn), *A. sullivantii* Engelm. ex A. Gray (prairie milkweed), *A. verticillata* L. (whorled milkweed), and several others. Because the relationship between milkweeds and AM fungi remains largely unexplored, many knowledge gaps exist, including the direction and magnitude of feedbacks occurring between sympatric species. Filling these gaps can provide insight into intraspecific organization on landscapes where congeners co‐occur, and how these dynamics may be mediated through PSFs.

To assess the PSF dynamics between sympatric congeners, a greenhouse study was conducted to assess the strength and direction of PSFs occurring between a common, clonal milkweed species [*A. syriaca* (focal species)] and four sympatric congeners across a gradient of increasing phylogenetic distances [*A. tuberosa*, *A. viridis*, *A. sullivantii*, and *A. verticillata*, respectively (collectively, peripheral species)]. The influence of *A. syriaca* on sympatric congeners, as well as the reciprocal effects, were measured by comparing biomass production and AM fungal colonization of peripheral species grown in *A. syriaca*‐ and conspecific‐conditioned soils. Due to previous research suggesting many native plant species perform poorly in soil conditioned by conspecifics, we hypothesize that our selected milkweed species will produce greater biomass and have greater AM fungal colonization in soil conditioned by heterospecifics (i.e. negative PSF). Furthermore, we hypothesize that this difference will be amplified as the phylogenetic distance of the “conditioning” species increases. Native plants often promote negative PSFs, although previous research suggests positive or neutral PSFs may be observed between closely related species (Liu et al., 2011; Crawford et al., 2019). Because of this, we expect neutral or weakly negative PSFs between *A. syriaca* and more closely related species, such as *A. tuberosa* and *A. viridis*, with PSFs becoming more strongly negative as the phylogenetic distance increases, as with *A. sullivantii* and *A. verticillata*. Examination of PSFs of closely related species is largely non‐existent, including species at the center of current conservation concerns, such as *Asclepias* spp. By examining the above‐ and belowground effects (i.e. biomass production and AM fungal colonization, respectively) of PSFs between co‐occurring congeners, we may better understand the role of plant–soil–microbial interactions in shaping plant communities. In addition, this research can provide key insight into the coexistence dynamics of related species on the landscape, as well as the degree to which evolutionary history dictates plant–soil–microbial interactions.

## 2 METHODS

### 2.1 Plant species, soil collection, and soil conditioning

In our experiment, we tested the plant–soil feedback dynamics of a focal plant species (*Asclepias syriaca*) and four sympatric congeners spanning across a phylogenetic gradient. *Asclepias syriaca* is a long‐lived perennial, clonal milkweed species native to much of eastern and central North America. The four peripheral species, from decreasing to increasing phylogenetic distance from *A. syriaca*, were *A. tuberosa* (phylogenetic distance: 480.5), *A. viridis* (1155.0), *A. sullivantii* (1308.8), and *A. verticillata* (2441.1). Phylogenetic distances were derived from recent phylogenies constructed by Fishbein et al. (2018). Native tallgrass prairie soil was collected from the Konza Prairie Biological Station (KPBS), Manhattan, KS, USA, where all five species used in this experiment can be commonly found. Soil was sieved through a 10 mm sieve to remove rocks and coarse plant material.

All seeds for this experiment were purchased from Prairie Moon Nursery (Winona, MN, USA). In the initial phase of the experiment, soils were conditioned, by growing individual *Asclepias* seedlings in 650 ml dee‐pots (Stuewe & Sons, Inc., Tangent, OR, USA) containing 600 g of native prairie soil. Each conditioning pot contained a single seedling of one of the five species used in the experiment, and this process was repeated for all species. Plants were grown and maintained for 18 weeks, at which time the majority of individuals were beginning to senesce, and soil was collected for use as inoculum in a subsequent feedback experiment. For our focal species, *A. syriaca*, 80 seedlings were grown for the purposes of soil conditioning, whereas 20 individuals of each of the other four species were grown. This ensured that enough inoculum would be produced for each pairwise feedback between being tested. The soil conditioning phase consisted of 160 pots [(4 species × 20 replicates) + (1 species × 80 replicates)]. Conditioned soils were pooled by species and homogenized prior to use as inoculum for the PSF experiment.

### 2.2 Plant–Soil feedback (PSF) experiment

The experimental design of the PSF test follows the feedback approach described by Bever (1994). Seeds of all five species were germinated in moist vermiculite following 30 days of cold‐moist stratification. After two pairs of true leaves had formed (~24 days), individual seedlings were transplanted into pots (Stuewe & Sons, Inc., Tangent, OR, USA) filled with 600 g (dry weight) of soil partitioned into three layers: 400 g of steam‐pasteurized prairie (KPBS) soil [80°C for 2 h and allowed to cool for 72 h to eliminate biotic communities but retain soil abiotic traits (Hetrick et al., 1990, Wilson & Hartnett, 1998)], followed by 100 g of soil inoculum (described above in soil conditioning phase), followed by 100 g of steam‐pasteurized soil to protect from cross‐contamination during the growing period (Duell et al., 2019). For our focal species, *A. syriaca*, a single seedling was planted per pot and inoculated with soil conditioned by a conspecific or one of each of the four heterospecific peripheral species. For each of the four peripheral species, a single seedling was planted per pot and inoculated with soil conditioned by conspecifics or soil conditioned by *A. syriaca*. The feedback test consisted of a partially factorial design with 130 pots [(1 species × 5 inocula × 10 replicates) + (4 species × 2 inocula × 10 replicates)].

After 20 weeks, prior to shoot senescence, plants were harvested, and root and shoot biomass was separated. All biomass was dried at 60°C for 48 h and weighed. Roots were washed free of soil and three subsamples from each individual plant were collected and stained with Trypan blue to determine the percent mycorrhizal root colonization. AM fungal root colonization was scored by using the magnified gridline intersect method (McGonigle et al., 1990), using a compound microscope (200×) to measure the percentage of root colonized by AM fungal structures (hyphae + vesicles + arbuscules). For each root sample, three random sections of root length were scored separately, with a total of approximately 900 grids observed per sample. Reported colonization values of each sample represent the mean of these three subsets.

### 2.3 Statistical analyses

Feedbacks were calculated for total biomass and intraradical AM fungal colonization. Interaction coefficients were calculated to quantify PSF dynamics between *A. syriaca* and several peripheral congeners grown with inoculum conditioned by either conspecific or heterospecific plants. We used the following equation: *I*_*s*_ = G(*A*)_*α*_ − G(*A*)_*β*_ − G(*B*)_*α*_ + G(*B*)_*β*_, where *I*_*s*_ is the feedback interaction coefficient, G(*A*)_*α*_ is the growth of plant species *A* inoculated with conspecific soil, G(*A*)_*β*_ is the growth of plant species *A* inoculated with heterospecific soil, G(*B*)_α_ is the growth of plant species *B* inoculated with heterospecific soil and, *G*(*B*)_*β*_ is the growth of plant species *B* inoculated with conspecific soil (Bever et al., 1997). When *I*_*s*_ values are positive (*I*_*s*_ > 0), a net positive feedback on plant growth is generated by the soil community, and coexistence between plant species does not occur. Conversely, when *I*_*s*_ values are negative (*I*_*s*_ < 0), a net negative feedback on plant growth is generated by the soil community, and coexistence between plant species does occur (Bever, 2003). Interaction coefficient values were calculated for each combination of *A. syriaca* and the four peripheral species.

Using PROC‐GLM in SAS, we constructed a general linear model using log‐transformed (for normalization of data) biomass data and logit‐transformed colonization data as the dependent variables. Conditioning species identity and experimental species identity were used as factors with interactions for each focal‐peripheral species pairing. Plant–soil feedback was tested within the conditioning and experimental plant–species interaction, where conditioning species represents the plant species used in the conditioning phase of the experiment and experimental species refers to the plant being assessed in the second experiment (Bever, 1994). These analyses were conducted using the PROC‐GLM procedure in (SAS Institute, Cary, NC, U.S.A.), version 9.4 of the SAS System for Windows.

Prior to analysis, all data were tested for normality and homogeneity of variances using the Shapiro–Wilk and Levene's tests, respectively. Lastly, simple linear regressions were constructed to test the effect of pairwise phylogenetic distance on PSF dynamics. These analyses were performed in R version 4.0.2 (R Core Team, 2020).

## 3 RESULTS

**FIGURE 1:**
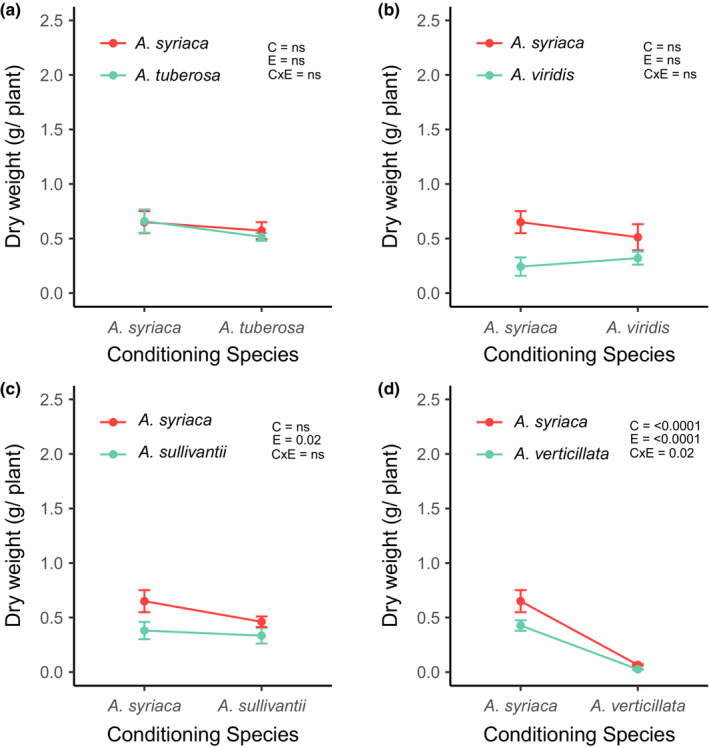
Biomass production of *A. syriaca* and paired peripheral species across a phylogenetic gradient. Phylogenetic distance from *A. syriaca* increases from panels a–d, with *A. tuberosa* and *A. verticillata* being the most closely and distantly related to *A. syriaca*, respectively. Within each panel, red points and lines represent *A. syriaca*, and green points and lines represent peripheral species. In the upper right‐hand of each panel, the effects of conditioning species (c), experimental species [E (i.e. identity of species grown during the experimental phase of the feedback experiment)], and the interaction of the two (CxE) are shown as represented by model *p*‐values, with significance assessed at *p* ≤ .05.

**FIGURE 2:**
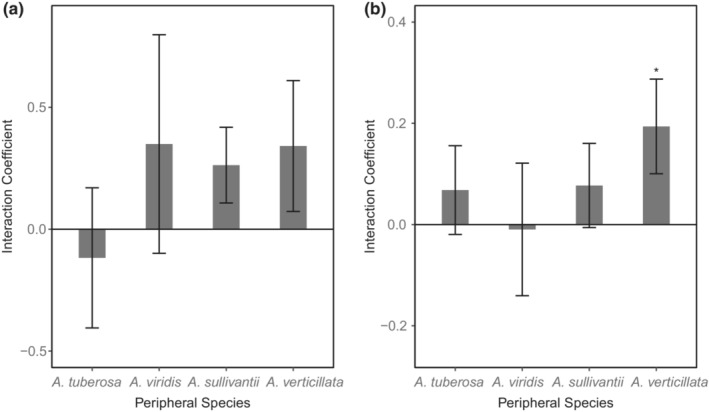
Plant–soil feedback (PSF) effects on (a) biomass production and (b) colonization by arbuscular mycorrhizal (AM) fungi of *Asclepias syriaca* and four sympatric congeners spanning a phylogenetic gradient. Within each panel, the phylogenetic distance from *A. syriaca* increases from left to right. Interaction coefficients were calculated using the following equation: *I*_*s*_ = G(a)α − G(a)β − G(b)α + G(B)β, where *I*_*s*_ is the feedback interaction coefficient, G(a)α is the growth of plant species A inoculated with conspecific soil, G(a)β is the growth of plant species A inoculated with heterospecific soil, G(b)α is the growth of plant species B inoculated with heterospecific soil and, G(B)β is the growth of plant species B inoculated with conspecific soil (Bever et al., 1997). Plant–soil feedbacks that differ statistically from zero are denoted by asterisks.

Biomass production of *A. syriaca* was not different among soil inoculum, with the exception of inoculum conditioned by *A. verticillata* (Figure 1d; Figure S1). In soils conditioned by *A. verticillata*, *A. syriaca* produced significantly less biomass compared to conspecific‐conditioned soils, as well as soils conditioned by *A. tuberosa*, *A. viridis*, and *A. sullivantii*. Biomass production of three of the four peripheral species was not different between *A. syriaca*‐ and conspecific‐conditioned soils, with the exception being *A. verticillata* (Figure 1d; Figure S2). Interestingly, *A. verticillata* produced significantly less biomass in conspecific‐conditioned soils, relative to soil conditioned by *A. syriaca* (Figure 1d; Figure S2). Interaction coefficients in biomass production tended to become more positive with increasing phylogenetic distance, but none of these individual feedbacks between *A. syriaca* and peripheral species were significant (Figure 2a).

**FIGURE 3:**
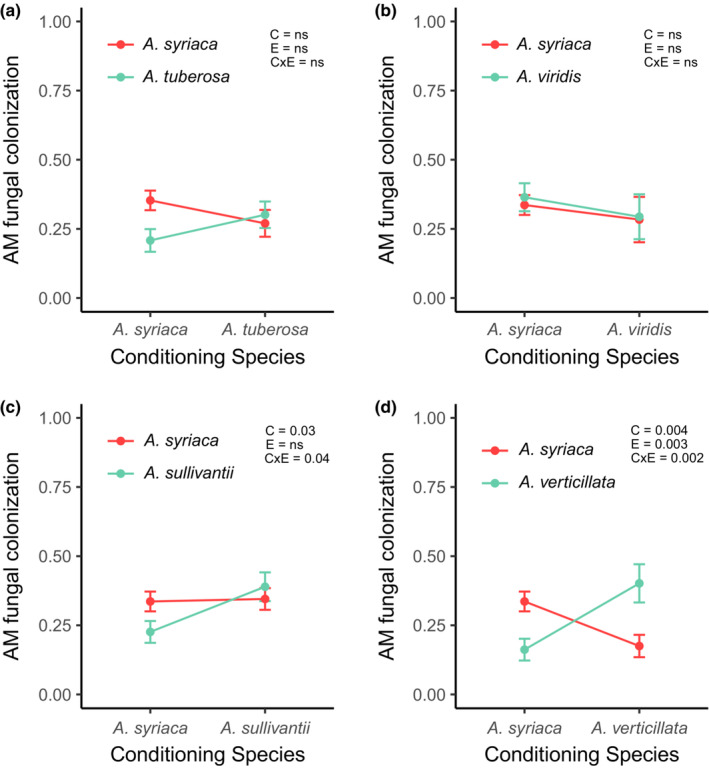
Proportion of intraradical arbuscular mycorrhizal (AM) fungal colonization of *A. syriaca* and paired peripheral species across a phylogenetic gradient. Phylogenetic distance from *A. syriaca* increases from panels a–d, with *A. tuberosa* and *A. verticillata* being the most closely and distantly related to *A. syriaca*, respectively. Within each panel, red points and lines represent *A. syriaca*, and green points and lines represent peripheral species. In the upper right‐hand of each panel, the effects of conditioning species (c), experimental species [E (i.e. identity of species grown during the experimental phase of the feedback experiment)], and the interaction of the two (CxE) are shown as represented by model *p*‐values, with significance assessed at *p* ≤ .05.

Overall, there were no significant differences in AM fungal colonization among *A. syriaca* grown in conspecific‐ or heterospecific‐conditioned soils (Figure 3; Figure S3). However, similar to patterns seen in biomass production, AM fungal colonization was generally lower in *A. syriaca* plants grown in *A. verticillata*‐conditioned soils, relative to soils conditioned by *A. tuberosa*, *A. viridis*, and *A. sullivantii* (Figure 3a–d; Figure S3). In fact, when grown in *A. verticillata*‐conditioned soils, *A. syriaca* displayed significantly lower AM fungal colonization levels, relative to conspecific‐conditioned soils (Figure 3d). Interestingly, as *A*. *verticillata* is the most phylogenetically distant peripheral species, our observation of greater colonization in conspecific‐conditioned soil with the *A. verticillata*–*A. syriaca* pairing is qualitatively consistent with greater mycorrhizal specialization with greater phylogenetic distance (Figures 2, 3),

## 4 DISCUSSION

We investigated the PSF and AM fungal dynamics between a common milkweed species (*A. syriaca*) and several sympatric congeners spanning a phylogenetic gradient (*A. tuberosa*, *A. viridis*, *A. sullivantii*, and *A. verticillata*, respectively). Overall, two clear patterns emerged. First, biomass PSF between *A. syriaca* and all four peripheral species were generally weakly positive. Secondly, AM fungal interaction coefficients indicate greater colonization density when milkweeds were grown with conspecific soils, as is consistent with AM fungal specialization on plant species, and this was particularly true for the two more phylogenetically distant species pairs (*A. syriaca* with *A. sullivantii* and *A. verticillata*, respectively). Such specialization of AM fungi would be expected to generate positive PSF and, though not significant, the PSF tended to be most positive between the two most distantly related species, *A. syriaca* and *A. verticillata*. With an estimated 400 *Asclepias* found throughout North America, parts of South America, and sub‐Saharan Africa, the inclusion of additional species would certainly strengthen this type of experiment; however, as we were interested primarily in local‐scale community dynamics including sympatric congeners, the number of available species was limited.

Phylogenetically‐driven PSF is often linked to the presence and abundance of host‐specific pathogens (Collins et al., 2020; Gilbert & Webb, 2007; Parker et al., 2015) or mutualists (Bever, 2003; Mangan, Herre, & Bever, 2010; Wang et al., 2017), and the degree to which the associated microbiomes of the two plants overlap. Interestingly, significant results in our study centered around the pairwise interactions between *A. syriaca* and *A. verticillata*, the most phylogenetically distant of the four peripheral species. For example, we found no differences in the productivity of *A. syriaca* among conditioned soils, with the exception of soil conditioned by *A. verticillata*. These results suggest the reduced productivity of *A. syriaca* in *A. verticillata*‐conditioned soils is likely driven by a lack of shared mutualists, namely AM fungi. Similar patterns have been observed in tropical rainforests, where seedlings of tree species performed better in soils and AM fungal communities conditioned by conspecifics, compared to those of heterospecific trees (Mangan, Herre, & Bever, 2010). Alternatively, the only peripheral species to be affected by soil conditioning was *A. verticillata*, which produced greater biomass in soils previously occupied by *A. syriaca*, relative to conspecific‐conditioned soils, suggesting a build‐up of host‐specific pathogens. Taken together, our results suggest that PSF dynamics between *A. syriaca* and *A. verticillata* may be driven by both mutualists and pathogens simultaneously, with mutualist dynamics likely having the strongest influence on *A. syriaca* productivity, and pathogen load‐driving patterns observed in *A. verticillata*. While the effects of phylogenetic distance on PSF dynamics have been observed in other species pairings (Crawford et al., 2019), these patterns have rarely been observed at such fine phylogenetic scales. Although no interactions or significant PSFs were detected between *A. syriaca* and the three more closely related peripheral species, significantly greater AM fungal colonization with conspecific‐conditioned soils between the most distantly related pairs of species does not rule out the possibility of a phylogenetic threshold. However, it is also possible other factors are at play, including, but not limited to, host life cycle, CSR (competitor, stress‐tolerator, ruderal) strategies, or the degree of responsiveness to symbionts (Davison et al., 2020; Koziol et al., 2022).

While most PSF studies assess the influence of plants, soils, and reciprocal interactions on subsequent biomass production, the flexibility of the interaction coefficient calculation allows for the testing of differential impacts of conspecific vs heterospecific inocula on a number of quantifiable plant traits. In our study, AM fungal root colonization of our focal species did not differ, regardless of conditioning species identity. Interestingly, *A. tuberosa*, the most closely related to our focal species, displayed a weakly greater AM fungal colonization in conspecific inocula. However, AM fungal colonization of *A. sullivantii* and *A. verticillata* was much greater in conspecific‐conditioned soils, compared to *A. syriaca*‐conditioned soils, with significantly positive interaction coefficients. These results suggest an absence of mutualists in soils conditioned by *A. syriaca* (Bever, 2002), which may be attributed to the phylogenetic distances of these species. Overall, these results are in line with several previous studies that have found greater mutualist abundances in soils previously occupied by conspecifics (Haskins & Gehring, 2005; Kulmatiski et al., 2017; Wang, Hoffland, et al., 2019; Wang, Jiang, et al., 2019).

The positive AM fungal interaction coefficients seen in our study may be due at least in part to the clonal nature of some of our study species. For clonality to be a successful form of asexual reproduction and lateral spread, appropriate soil conditions must exist for the establishment of new ramets. Negative PSF has been observed in several clonal plant species and is thought to be a driving mechanism of ring formation in some species (Bonanomi et al., 2014; Cartenì et al., 2012). The connectedness of rhizomes or stolons may increase susceptibility to soil pathogens, and recent evidence suggests that some stoloniferous grasses perform better in sterile soil, relative to soils previously occupied by conspecifics, though these results may be species‐specific (Xue et al., 2022). Alternatively, extensive rhizomes may facilitate pathogen escape, thus weakening the effect of negative PSF (D'Hertefeldt & van der Putten, 1998). It is worth noting that we did not test mother plants and ramets due to time constraints, as this would take multiple years to grow plants to maturity. While it is possible that plant ontogeny (e.g. seedling vs. ramet) influences PSF outcomes, this area of work remains largely unexplored. Three out of five of our *Asclepias* species (*A. syriaca*, *A. sullivantii*, and *A. verticillata*) exhibit clonality, and the two clonal peripheral species (*A. sullivantii* and *A. verticillata*) consistently exhibited the strongest, albeit not always significant, positive interaction coefficients. Although there was no significant effect of conditioning species identity on AM fungal colonization of *A. syriaca*, both *A. sullivantii* and *A. verticillata* exhibited significantly greater AM fungal colonization in conspecific‐conditioned soils, indicating a presence of selected mutualists in conspecific‐conditioned soils. However, greater colonization levels did not necessarily translate to conferred benefits, as *A. verticillata* produced very little biomass in conspecific‐conditioned soils, highlighting the need for further investigation of *Asclepias*–microbial interactions and specificity (Koziol et al., 2022).

Our study provides novel insight into the effects of plant–soil interactions on biomass production and AM fungal colonization in coexisting *Asclepias* species, yet many knowledge gaps remain. We assessed PSF dynamics between a single, focal species and several peripheral congeners. Even within the same genus, it has been well‐established that plants can vary widely in their responses to, and selection of, AM fungal partners (Koziol et al., 2022; Wilschut et al., 2019), making microbial community abundances and assemblages crucial for understanding plant‐microbial dynamics among related species. In the context of our study, it is possible that pairwise PSFs between our peripheral species may differ in direction and magnitude, depending on the degree of host specificity of mutualistic and pathogenic communities. With the rapid advancement in molecular tools and techniques, researchers are able to identify AM fungal taxa in soils. This, along with emerging interest in host‐symbiont specificity within plant communities (Klironomos, 2002; Sepp et al., 2018; Wang, Hoffland, et al., 2019; Wang, Jiang, et al., 2019), provides a unique opportunity to assess differences in microbial community composition between and among related species. As plant community succession advances temporally, species within those communities often display greater degrees of host specificity with regard to AM fungal symbionts (Koziol & Bever, 2016; Cheeke et al., 2019). Successional stages of *Asclepias* are often species‐specific and context‐dependent (Bauer et al., 2018; Middleton & Bever, 2012), with many considered to be mid‐successional. Thus, it is likely that a certain degree of AM fungal host specificity exists within the genus, as supported by recent studies (Koziol et al., 2022).

Interactions between plants and their associated soils are recognized as key drivers of plant community diversity and organization on the landscape (Bever et al., 2015; Mangan, Schnitzer, et al., 2010) and may be especially important for sympatric‐related species (Mangan, Herre, & Bever, 2010). While recent literature suggests that pairs of distantly related species likely exhibit more negative PSF due to pathogen specialization (Crawford et al., 2019; Mangan, Herre, & Bever, 2010), these patterns may occasionally be masked by the absence of shared mutualists, resulting in stronger positive feedbacks. Our results suggest PSF dynamics between *Asclepias* species are likely driven by mutualist communities. In short, our data indicate that soil conditioning by heterospecific congeners generally does not affect productivity, regardless of phylogenetic distance. However, this was not the case for paired phylogenetically distant species (Crawford et al., 2019). Furthermore, our study indicates that sympatric *Asclepias* species are capable of conditioning soils to the benefit of conspecifics with regard to intraradical AM fungal abundances, but that these results do not translate to plant productivity metrics such as total biomass production. While our study consisted of four sympatric congeners, further research is warranted to determine whether this is a generalizable pattern across additional *Asclepias* species. Phylogenetic signals within and among plant–microbial interactions are well‐documented, though empirical evidence with a focus on congeners is severely lacking. Our research provides support for change in directionality and magnitude of PSF across congeneric phylogenetic distances and provides a baseline for further similar studies.

## AUTHOR CONTRIBUTIONS

**Eric Duell:** Conceptualization (equal); data curation (lead); formal analysis (equal); investigation (lead); methodology (lead); project administration (equal); software (equal); validation (lead); visualization (lead); writing – original draft (lead); writing – review and editing (lead). **James D. Bever:** Formal analysis (equal); methodology (supporting); software (equal); validation (equal); writing – review and editing (equal). **Gail Wilson:** Funding acquisition (lead); project administration (supporting); resources (lead); supervision (lead); writing – review and editing (equal).

## CONFLICT OF INTEREST

The authors declare no competing interests.

## Supporting information


**Figure S1.** **–S4.**

## Data Availability

Data will be uploaded in the Dryad upon acceptance.
