# Supplementary material for: Role of plant relatedness in plant–soil feedback dynamics of sympatric Asclepias species
Source: Ecol Evol. 2023 Jan 24;13(1):e9763. doi: 10.1002/ece3.9763 (PMC9873585; doi:10.1002/ece3.9763)
Supplement: Supplementary file 1 — Figure S1. –S4. [file ECE3-13-e9763-s001.docx]

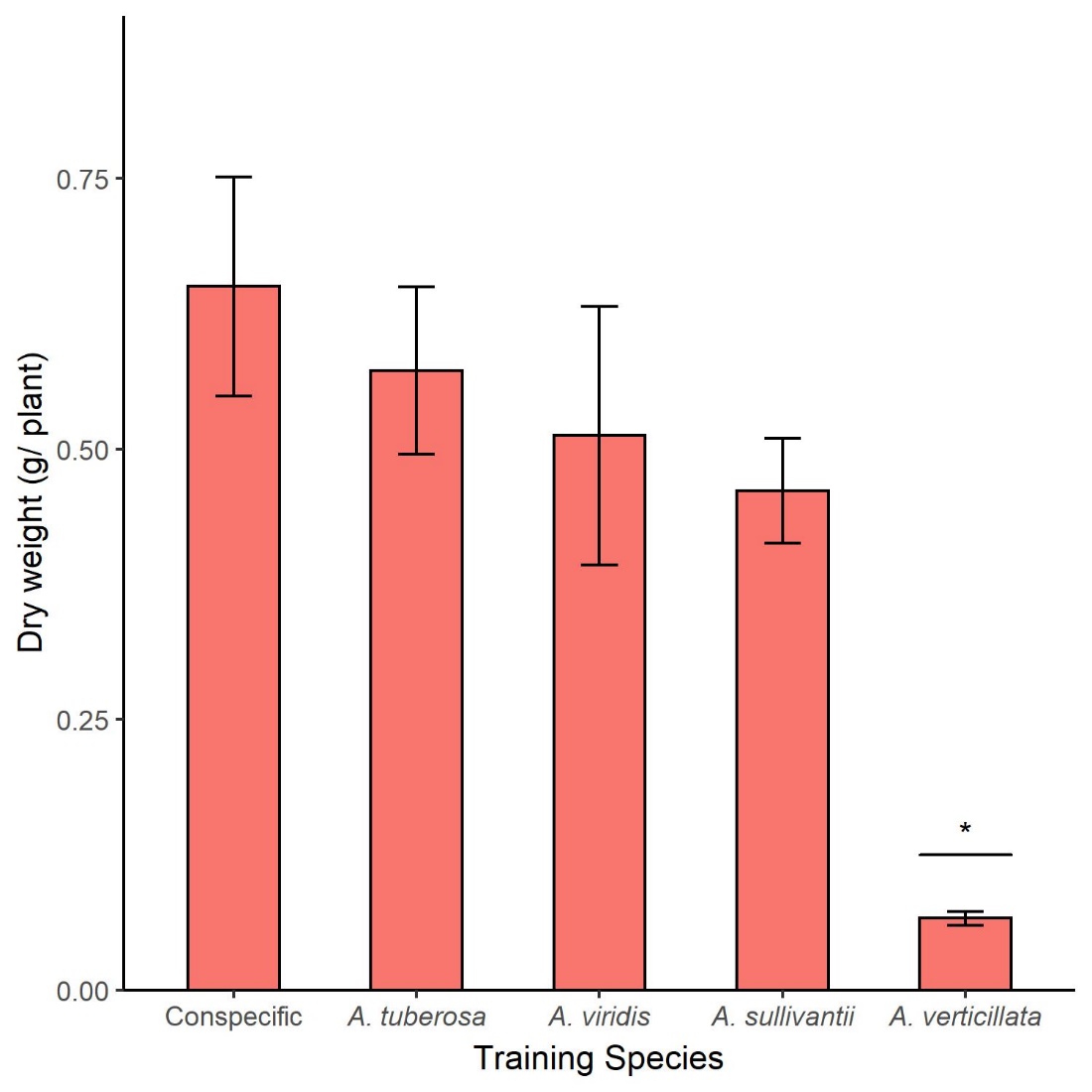


Figure S1. Biomass production of *A. syriaca* in response to soil conditioning by sympatric congeners spanning a phylogenetic gradient. Phylogenetic distance from *A. syriaca* increases from left to right.


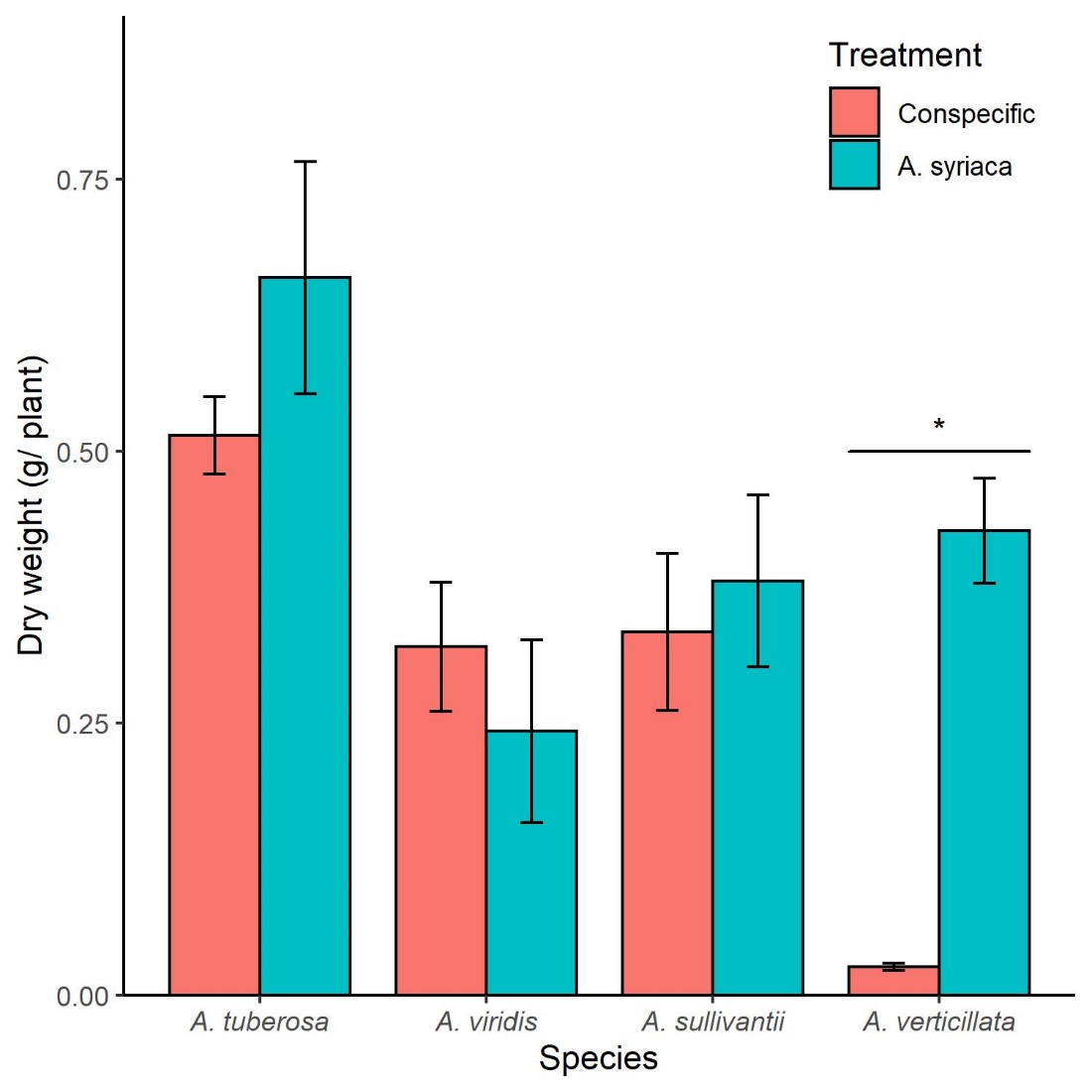


Figure S2. Biomass production of peripheral species in conspecific- (red bar) and *A. syriaca*-trained (blue bar) soils. Asterisks denote significant differences within a species, with significance assessed at p ≤ 0.05.


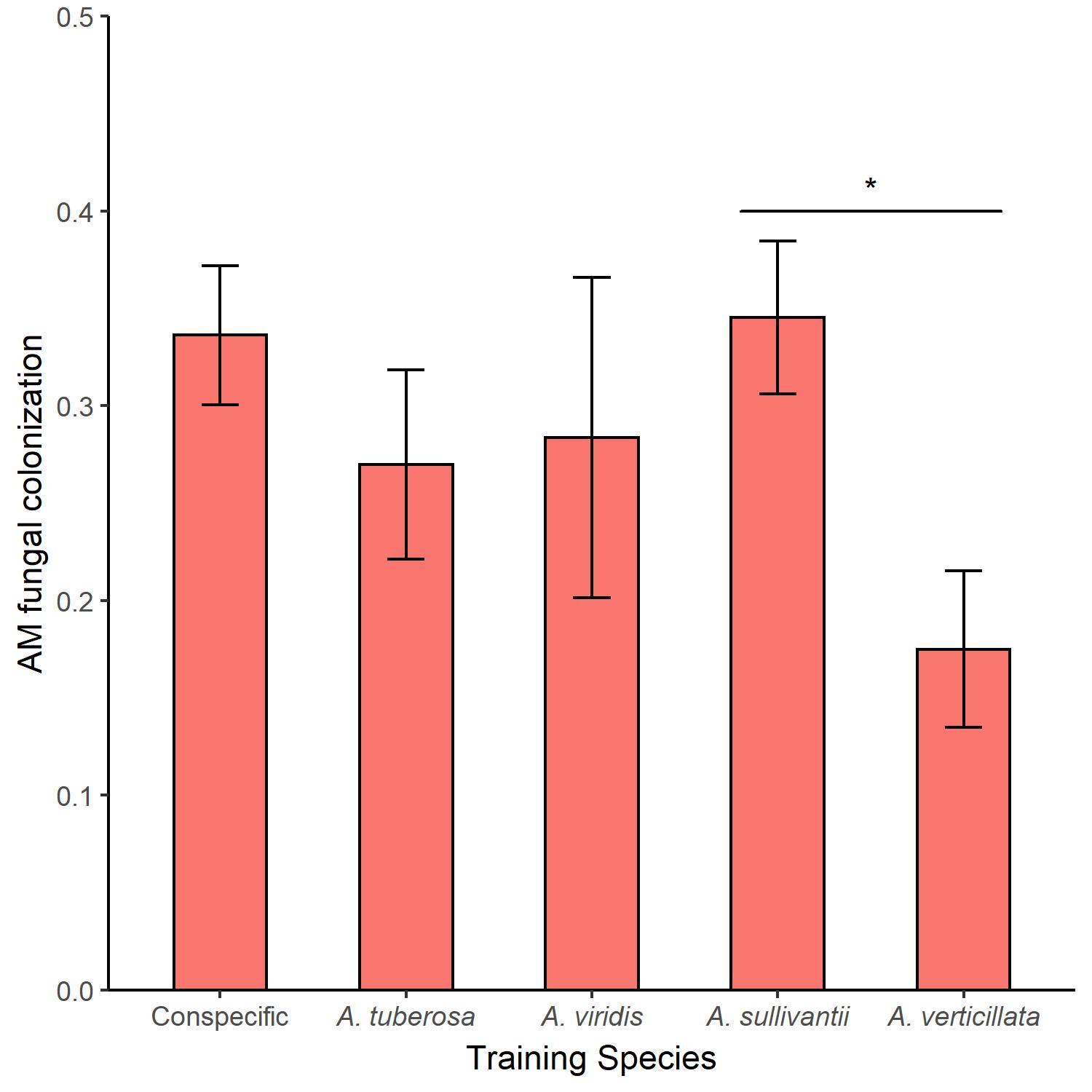


Figure S3. AM fungal colonization of *A. syriaca* in response to soil conditioning by sympatric congeners spanning a phylogenetic gradient. Phylogenetic distance from *A. syriaca* increases from left to right. Asterisks denote significant differences between training species treatments, with significance assessed at p ≤ 0.05.


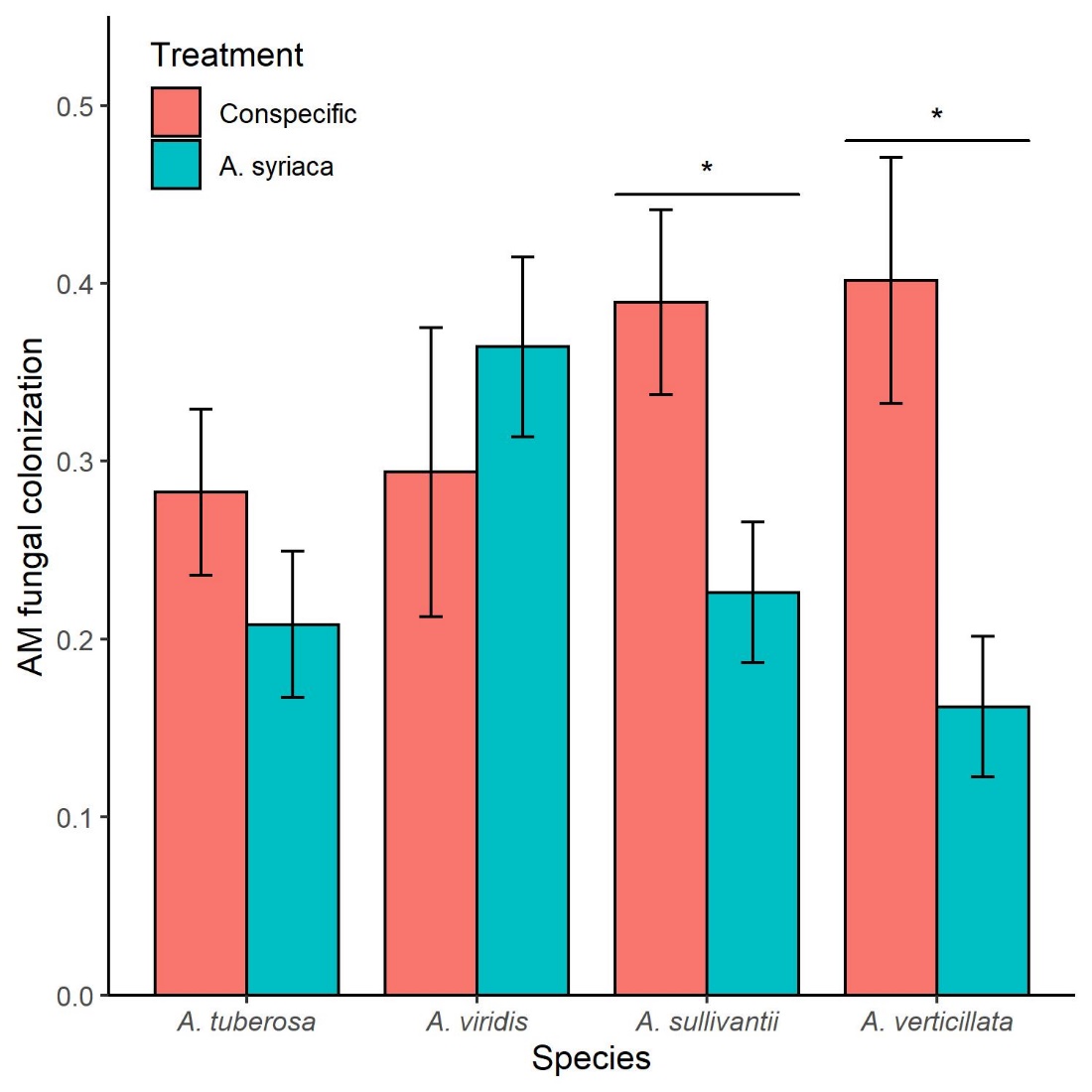


Figure S4. AM fungal colonization of peripheral species in conspecific- (red bar) and *A. syriaca*-trained (blue bar) soils. Asterisks denote significant differences within a species, with significance assessed at p ≥ 0.05.
